# Supplementary material for: Therapeutic strategy for spinal muscular atrophy by combining gene supplementation and genome editing
Source: Nat Commun. 2024 Jul 24;15:6191. doi: 10.1038/s41467-024-50095-5 (PMC11269569; doi:10.1038/s41467-024-50095-5)
Supplement: Supplementary file 1 — Supplementary Information [file 41467_2024_50095_MOESM1_ESM.pdf]

Supplementary Fig. 1

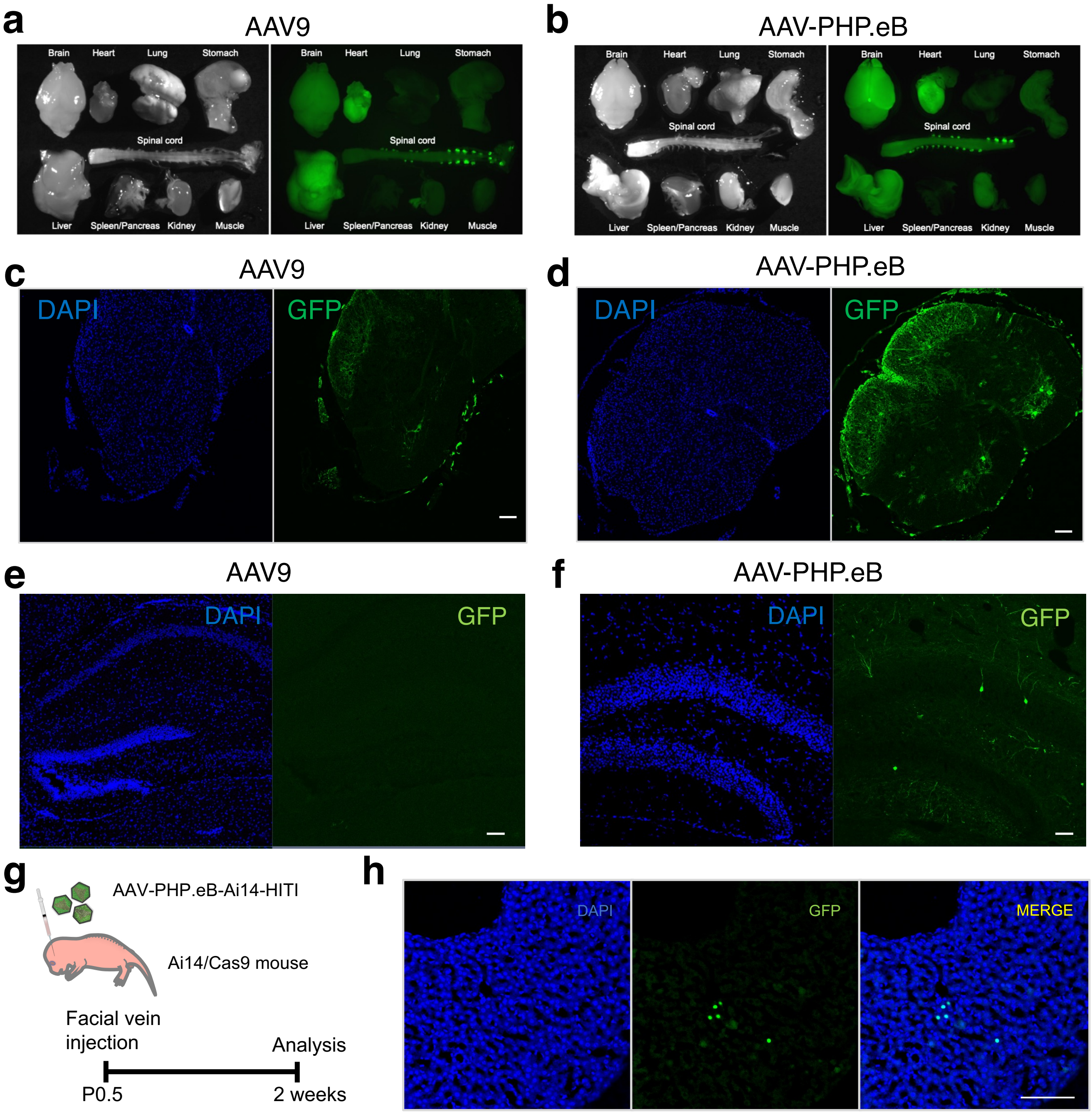

Supplementary Fig. 1. **Comparison of transduction in tissues between AAV9 and AAV-PHP.eB**

(**a**), GFP expression in the brain, heart, lung, stomach, spinal cord, liver, spleen, pancreas, kidney and muscle 14 days following facial vein injection in AAV9-GFP injected mouse. The GFP signal is primary in the heart, liver and dorsal root ganglion. (**b**), GFP expression in the brain, heart, lung, stomach, spinal cord, liver, spleen, pancreas, kidney and muscle 14 days following facial vein injection in AAV-PHP.eB-GFP injected mouse. The GFP signal is primary in the brain, heart, liver, spinal cord and dorsal root ganglion. (**c-f**), Immunostaining for GFP of spinal cord (**c** and **d**), hippocampus (**e** and **f**) in AAV9-GFP (**c** and **e**) or AAV-PHP.eB-GFP (**d** and **f**) injected mouse 14 days following facial vein injection. Scale bar, 100 μm. (**g**), AAV-PHP.eB-Ai14-HITI was systemically delivered via facial vein in neonatal Ai14-Cas9 mice and analyzed 2 weeks after the injection. (**h**), Representative immunofluorescence images of GFP in the AAV-injected liver sections. Scale bar, 100 μm.

Supplementary Fig. 2

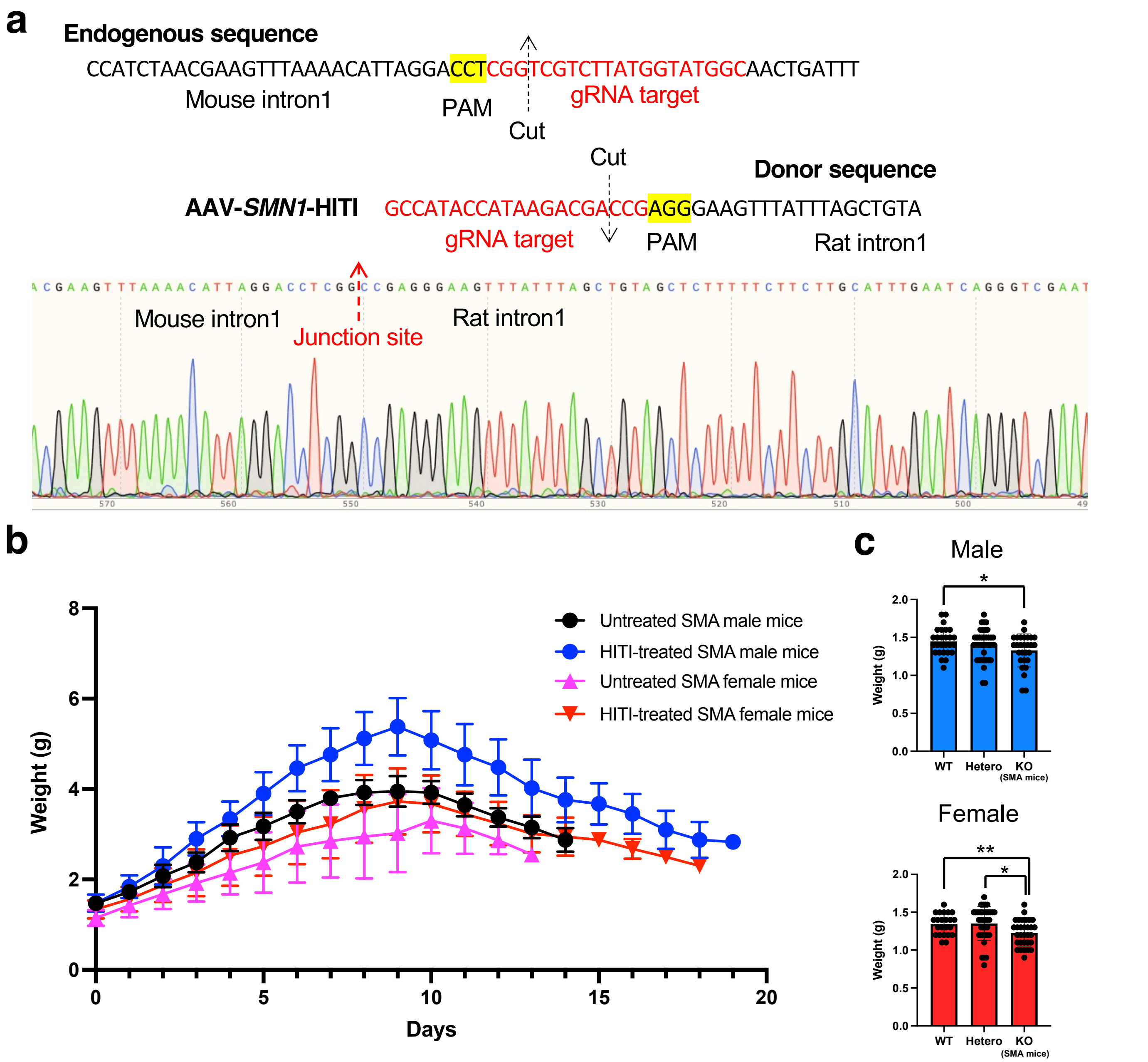

Supplementary Fig. 2. **HITI-mediated gene correction of a mouse model of SMA**

(**a**), Schematic of correct gene knock-in in HITI-treated SMA mice. Red color shows gRNA target sequences following the PAM sequences (yellow color). Black dotted arrow indicates Cas9 cleavage site. Sequence is validated on the bottom. (**b**), Body weight over time in untreated and HITI-treated SMA mice, which was assessed independently from the data shown in Fig. 2e and 2f. Male mice are indicated by circles, and female mice are represented by triangles. Untreated SMA mice are shown with black (male, n=4) and pink (female, n=4) lines, while HITI-treated SMA mice are represented by blue (male, n=5) and red (female, n=8) lines. (**c**), Body weight comparison between WT (*SMN2*$^{+/+}$; SMNΔ7$^{+/+}$; *Smn1*$^{+/+}$), heterozygous (*SMN2*$^{+/+}$; SMNΔ7$^{+/+}$; *Smn1*$^{+/-}$) and SMA (KO) mice (SMA mice, *SMN2*$^{+/+}$; *SMN*Δ7$^{+/+}$; *Smn1*$^{-/-}$) at P0.5 with males depicted on the top (WT, n=27; Hetero, n=38; KO, n=28), and females depicted on the bottom (WT, n=23; Hetero, n=37; KO, n=31). ***p*<0.01, **p*<0.05, a two-sided unpaired Student's *t*-test. Source data are provided as a Source Data file.

Supplementary Fig. 3

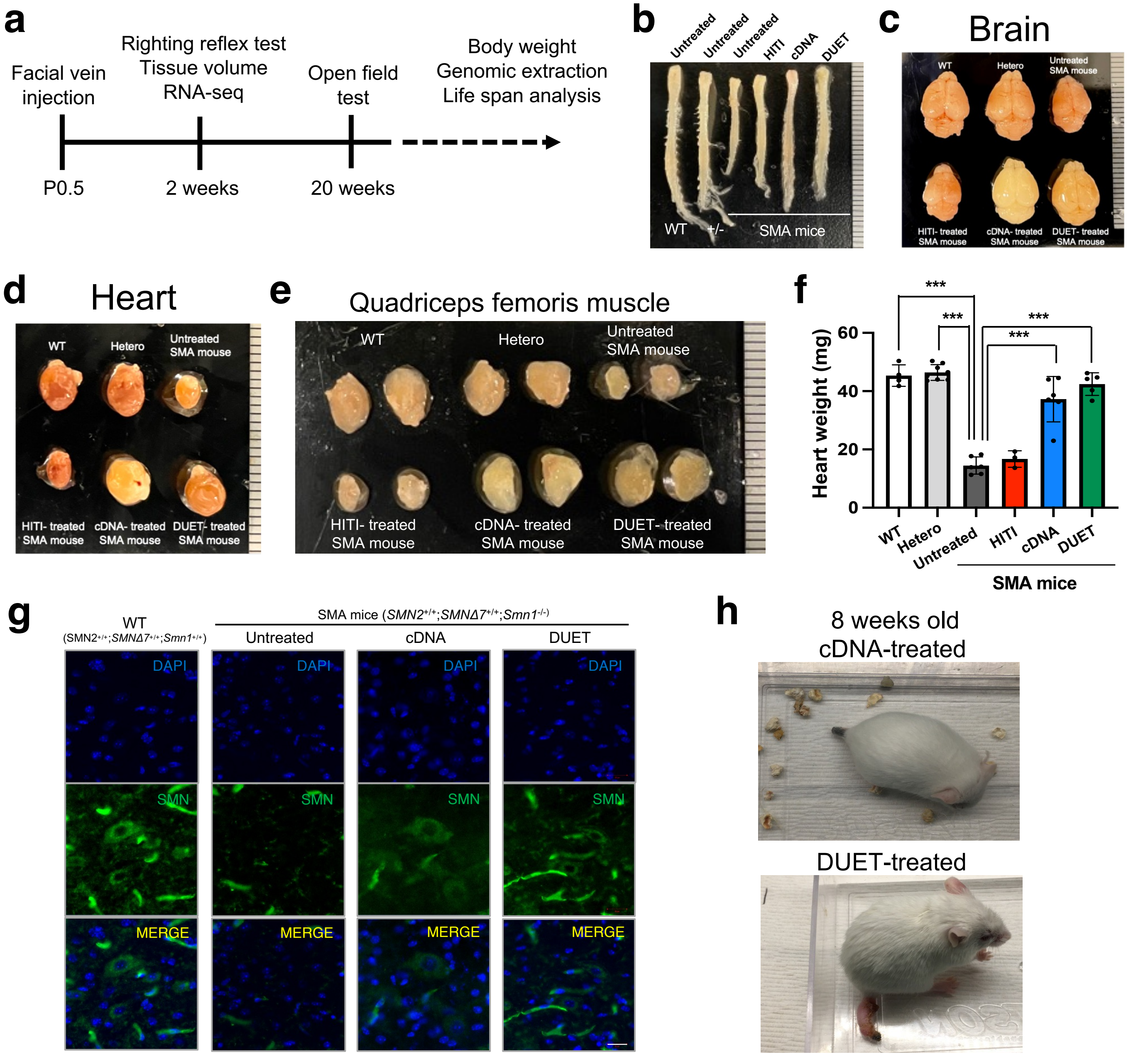

Supplementary Fig. 3. Gross morphology of treated SMA mice

(**a**), Body weight and life span were recorded after AAVs injections. Righting reflex test, tissues volume analyses and RNA-seq were performed 2 weeks after the injections. The open field test was performed 20 weeks after the injections. (**b**), Gross morphology of the spinal cords in female untreated WT, heterozygous mice and SMA mice with no treatment or each treatment at 2 weeks old. Scale bar, 1 mm. (**c-e**), Gross morphology of the brains (**c**), hearts (**d**) and quadriceps femoris muscles (**e**) in untreated male WT, heterozygous mice and SMA mice with no treatment or each treatment at 2 weeks old. Scale bar, 1 mm. (**f**), Weight of the heart in untreated WT mice (n=4), heterozygous mice (n=8) and SMA mice with no treatment (n=6) or HITI (n=3), cDNA (n=6) and DUET (n=5) treatments. Both cDNA and DUET treatments improved heart atrophy in SMA mice. ***p<0.0001, a two-sided unpaired Student's t-test (vs untreated SMA mice). (**g**), Representative immunofluorescence images of SMN in the spinal cord sections in WT, SMA mice with no treatment or each treatment. Scale bar, 20 μm. (**h**), Gross morphology of tail necrosis in treated SMA mice. Shown are 8 weeks old cDNA-treated SMA mouse on the top and DUET-treated SMA mouse on the bottom. Source data are provided as a Source Data file.

Supplementary Fig. 4

a

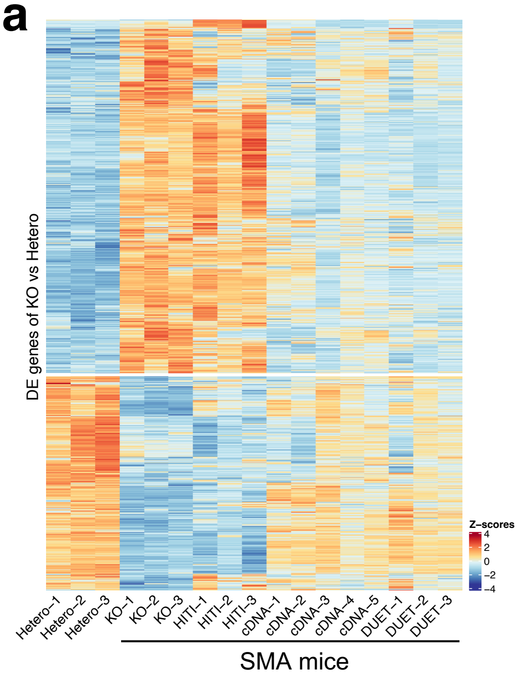

d

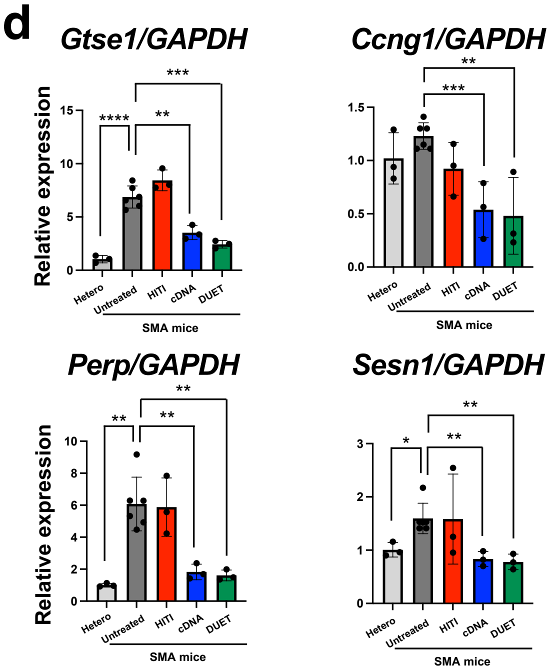

b **Upregulated biological process in SMA mice (vs Heterozygous mice)**

Description	Genes	*p* value	FDR
drug catabolic process	38	4.37E-14	3.55E-11
protein activation cascade	20	2.04E-10	8.27E-08
negative regulation of hydrolase activity	58	9.17E-08	2.48E-05
organic hydroxy compound metabolic process	57	2.68E-07	5.45E-05
regulation of peptidase activity	56	3.43E-07	5.57E-05
lipid catabolic process	41	6.93E-07	8.03E-05
organic anion transport	50	9.53E-06	5.53E-04
response to peptide	52	2.72E-05	8.89E-04
response to wounding	53	4.01E-05	1.16E-03
circulatory system process	49	1.55E-04	3.60E-03

c **Downregulated biological process in SMA mice (vs Heterozygous mice)**

Description	Genes	*p* value	FDR
monovalent inorganic cation transport	42	3.40E-10	2.76E-07
regulation of ion transmembrane transport	36	2.36E-08	9.59E-06
regulation of membrane potential	34	1.07E-07	2.90E-05
muscle system process	26	2.63E-05	5.34E-03
receptor-mediated endocytosis	20	5.30E-05	8.61E-03
neurotransmitter transport	21	1.23E-04	1.43E-02
microtubule-based movement	19	1.68E-04	1.52E-02
locomotory behavior	18	1.94E-04	1.58E-02
circulatory system process	28	2.43E-04	1.80E-02
sensory perception of mechanical stimulus	14	6.74E-04	3.65E-02

Supplementary Fig. 4. Analyses of RNA-seq
(**a**), Heatmap analysis of RNA-seq in the spinal cord from untreated heterozygous mice and SMA mice with no treatment or each treatment 2 weeks after the injections. (**b and c**), Biological process of upregulation (**b**) and downregulation (**c**) in SMA mice compared to heterozygous mice by gene-set enrichment analyses. (**d**), RT-qPCR analysis for the expression of *Gtse1*, *Ccng1*, *Perp* and *Sesn1* in the spinal cords from untreated heterozygous mice (n=3) and SMA mice with no treatment (n=6) or HITI (n=3), cDNA (n=3) and DUET (n=3) treatments. The expression level of each gene was normalized by *Gapdh* first, and then the ratio against heterozygous samples was calculated. Data are represented as mean ± S.D. **** $p<0.0001$, ***$p<0.001$, **$p<0.005$, *$p<0.05$, a two-sided unpaired Student's *t*-test. Source Data are provided as a Source Data file.

Supplementary Fig. 5

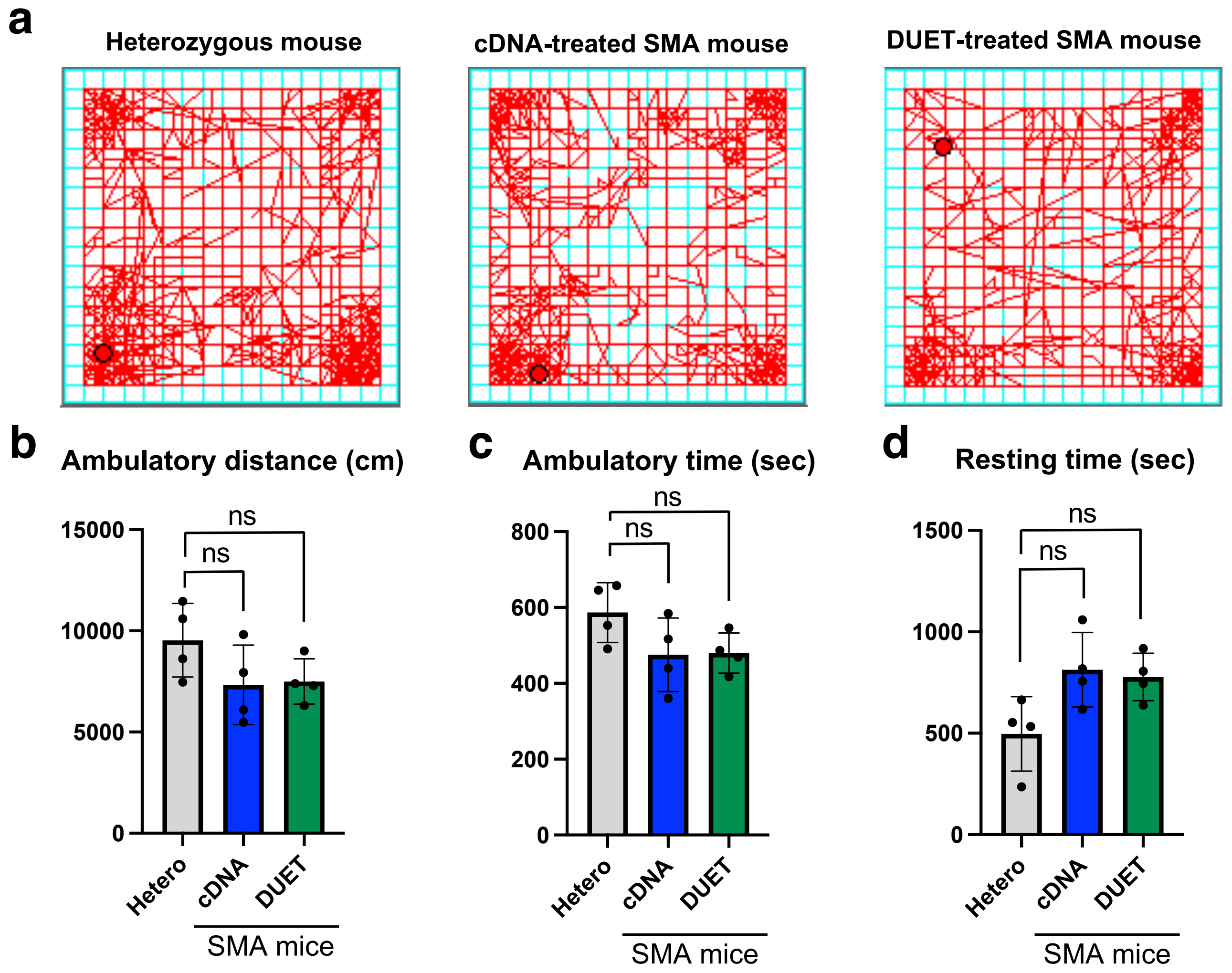

Supplementary Fig. 5. Locomotor activity of treated SMA mice of 20 weeks old
(**a**), Representative total trace image from untreated heterozygous mouse (n=4), SMA mouse with cDNA treatment (n=4) and DUET treatment (n=4). (**b**), Total ambulatory distance. (**c**), Total ambulatory time. (**d**), Total resting time. Each data point represents the total value in a 30 min. n= 4 for all mice. Data are represented as mean ± S.D. ns, not significant. A two-sided unpaired Student's *t*-test was performed. Source data are provided as a Source Data file.

Supplementary Fig. 6

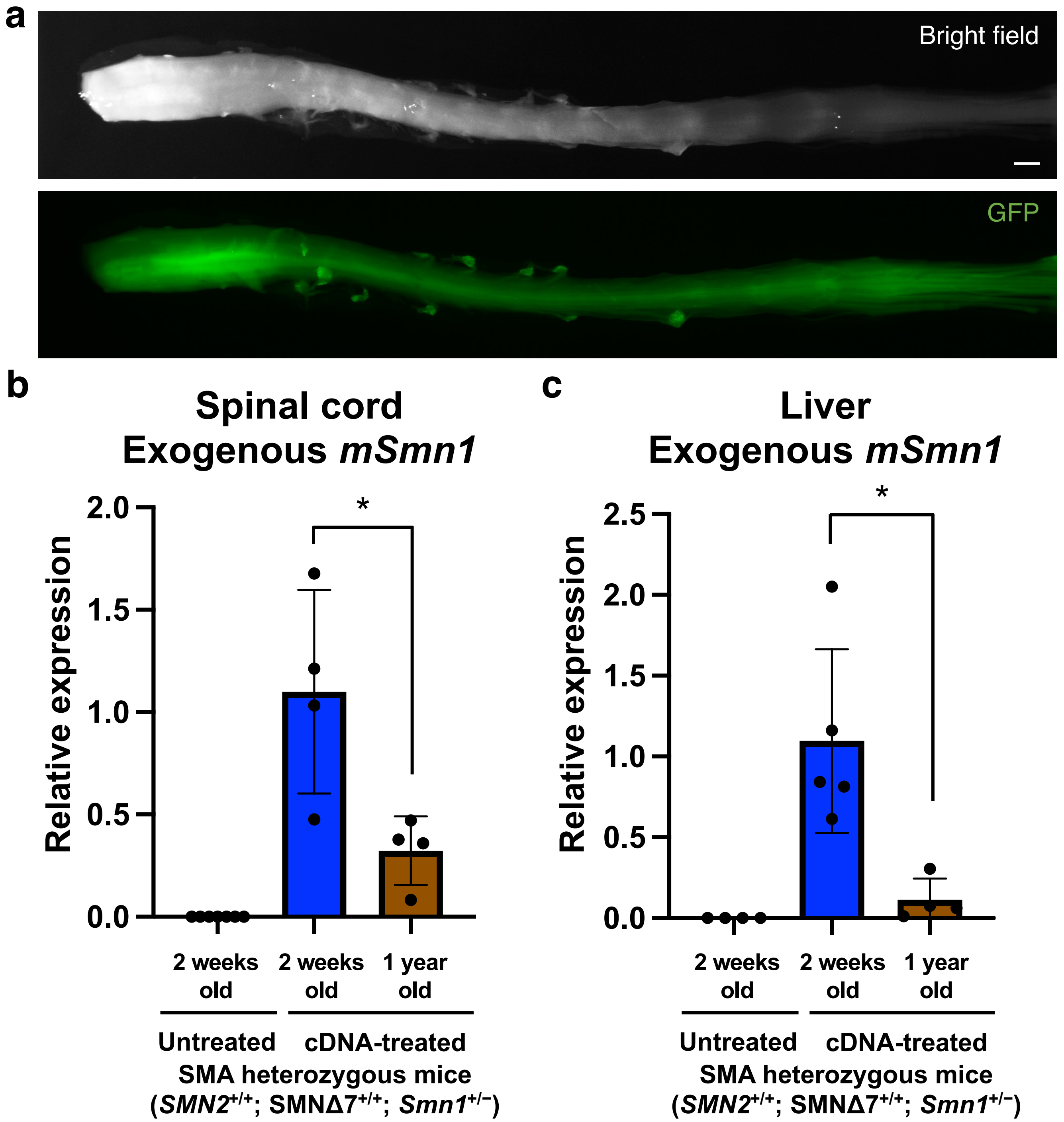

Supplementary Fig. 6. **Reduction of exogenous transgene by AAV in tissues 1 year later**
(**a**), Bright field (upper) and GFP (lower) image of the spinal cord in a 1 year old AAV-PHP.eB-GFP injected mouse. Scale bar, 1 mm. (**b and c**), RT-qPCR analysis for the expression of exogenous mouse *Smn1* (*mSmn1*) in the spinal cords (**b**) and livers (**c**) in untreated (Spinal cord, n=7; Liver, n=4) and cDNA-treated heterozygous mice 2 weeks (Spinal cord, n=4; Liver, n=5) or 1 year (Spinal cord, n=4; Liver, n=4) after the injections. The primers specifically detected the exogenous mouse *Smn1* from AAV because of no detection in untreated samples. The expression level of mouse *Smn1* was normalized by *Gapdh* first, and then ratio against 2 weeks old cDNA-treated samples was calculated. Data are represented as mean ± S.D. *p<0.05, a two-sided unpaired Student's t-test. Source data are provided as a Source Data file.
