# Supplementary material for: Therapeutic strategy for spinal muscular atrophy by combining gene supplementation and genome editing
Source: Nat Commun. 2024 Jul 24;15:6191. doi: 10.1038/s41467-024-50095-5 (PMC11269569; doi:10.1038/s41467-024-50095-5)
Supplement: Supplementary file 3 — Description of Additional Supplementary Files [file 41467_2024_50095_MOESM3_ESM.pdf]

**Description of Additional Supplementary Files**

**Supplementary Movie 1**. General behavior of untreated SMA mouse

**Supplementary Movie 2**. General behavior of HITI-treated SMA mouse

**Supplementary Movie 3**. General behavior of cDNA-treated SMA mouse

**Supplementary Movie 4**. General behavior of DUET-treated SMA mouse
