# Supplementary material for: Therapeutic strategy for spinal muscular atrophy by combining gene supplementation and genome editing
Source: Nat Commun. 2024 Jul 24;15:6191. doi: 10.1038/s41467-024-50095-5 (PMC11269569; doi:10.1038/s41467-024-50095-5)
Supplement: Supplementary file 8 — Reporting Summary [file 41467_2024_50095_MOESM8_ESM.pdf]

# nature portfolio

Corresponding author(s): Juan Carlos Izpisua Belmonte
Last updated by author(s): Jun 18, 2024

# Reporting Summary

Nature Portfolio wishes to improve the reproducibility of the work that we publish. This form provides structure for consistency and transparency in reporting. For further information on Nature Portfolio policies, see our Editorial Policies and the Editorial Policy Checklist.

## Statistics

For all statistical analyses, confirm that the following items are present in the figure legend, table legend, main text, or Methods section.

| n/a | Confirmed | |
|---|---|---|
| ☐ | ☒ | The exact sample size ($n$) for each experimental group/condition, given as a discrete number and unit of measurement |
| ☐ | ☒ | A statement on whether measurements were taken from distinct samples or whether the same sample was measured repeatedly |
| ☐ | ☒ | The statistical test(s) used AND whether they are one- or two-sided<br>*Only common tests should be described solely by name; describe more complex techniques in the Methods section.* |
| ☒ | ☐ | A description of all covariates tested |
| ☐ | ☒ | A description of any assumptions or corrections, such as tests of normality and adjustment for multiple comparisons |
| ☐ | ☒ | A full description of the statistical parameters including central tendency (e.g. means) or other basic estimates (e.g. regression coefficient) AND variation (e.g. standard deviation) or associated estimates of uncertainty (e.g. confidence intervals) |
| ☐ | ☒ | For null hypothesis testing, the test statistic (e.g. $F$, $t$, $r$) with confidence intervals, effect sizes, degrees of freedom and $P$ value noted<br>*Give P values as exact values whenever suitable.* |
| ☒ | ☐ | For Bayesian analysis, information on the choice of priors and Markov chain Monte Carlo settings |
| ☒ | ☐ | For hierarchical and complex designs, identification of the appropriate level for tests and full reporting of outcomes |
| ☒ | ☐ | Estimates of effect sizes (e.g. Cohen's $d$, Pearson's $r$), indicating how they were calculated |

*Our web collection on statistics for biologists contains articles on many of the points above.*

## Software and code

Policy information about availability of computer code

| | |
|---|---|
| Data collection | An Olympus IX51 microscope was used for histology images. ZEISS Zen software (v2.3 blue edition) was used for confocal imaging. |
| Data analysis | Prism 9 (version 9.4.1) was used for performing statistical analyses, and FASTQC (Babraham Bioinformatics) was used for RNA-seq analysis. |

For manuscripts utilizing custom algorithms or software that are central to the research but not yet described in published literature, software must be made available to editors and reviewers. We strongly encourage code deposition in a community repository (e.g. GitHub). See the Nature Portfolio guidelines for submitting code & software for further information.

## Data

Policy information about availability of data

All manuscripts must include a data availability statement. This statement should provide the following information, where applicable:

- Accession codes, unique identifiers, or web links for publicly available datasets
- A description of any restrictions on data availability
- For clinical datasets or third party data, please ensure that the statement adheres to our policy

Data supporting the findings of this study are available within the paper and its supplementary information files. RNA-sequence data have been deposited in the Gene Expression Omnibus under the accession number GSE207181.

## Human research participants

Policy information about studies involving human research participants and Sex and Gender in Research.

| | |
|---|---|
| Reporting on sex and gender | None were used |
| Population characteristics | None were used |
| Recruitment | None were used |
| Ethics oversight | None were used |

Note that full information on the approval of the study protocol must also be provided in the manuscript.

# Field-specific reporting

Please select the one below that is the best fit for your research. If you are not sure, read the appropriate sections before making your selection.

- [x] Life sciences
- [ ] Behavioural & social sciences
- [ ] Ecological, evolutionary & environmental sciences

For a reference copy of the document with all sections, see nature.com/documents/nr-reporting-summary-flat.pdf

# Life sciences study design

All studies must disclose on these points even when the disclosure is negative.

| | |
|---|---|
| Sample size | The sample sizes for each experiment are indicated in the figure legends. Sample size was chosen based on the sample availability and statistic relevance. Sample size was at least n=3 independent biological replicates. |
| Data exclusions | No data were excluded. |
| Replication | Number of biological replicates are described in figure legends. All experiments reported in this study were repeated at least three independent times. All samples evaluated at the level of RNAseq were performed in more than triplicate and the sequencing was all successful. |
| Randomization | No randomization method was used in this study. Animals and samples were randomly allocated into experimental groups. |
| Blinding | No blinding was performed during in vivo experiment, tissue collections and statistical analysis. |

# Reporting for specific materials, systems and methods

We require information from authors about some types of materials, experimental systems and methods used in many studies. Here, indicate whether each material, system or method listed is relevant to your study. If you are not sure if a list item applies to your research, read the appropriate section before selecting a response.

## Materials & experimental systems

| n/a | Involved in the study | |
|---|---|---|
| | x | Antibodies |
| x | | Eukaryotic cell lines |
| x | | Palaeontology and archaeology |
| | x | Animals and other organisms |
| x | | Clinical data |
| x | | Dual use research of concern |

## Methods

| n/a | Involved in the study | |
|---|---|---|
| x | | ChIP-seq |
| x | | Flow cytometry |
| x | | MRI-based neuroimaging |

## Antibodies

| | |
|---|---|
| Antibodies used | Anti-GFP (Aveslabs, GFP-1020), Anti-NeuN (EnCor Biotechnology Inc, MCA-1B7), Anti-ChAT (Novus Biologicals,NB110-89724), Anti-SMN, (610647, BD Biosciences). |
| Validation | Information from manufacture website was used for selecting the antibody. Anti-GFP was validated in previous studies. (Nature, 540, 144-149,2016). Anti-NeuN was validated in mouse brain and spinal cord samples. Anti-SMN was validated in mouse brain and spinal |

cord samples.

## Animals and other research organisms

Policy information about studies involving animals; ARRIVE guidelines recommended for reporting animal research, and Sex and Gender in Research

| | |
|---|---|
| Laboratory animals | C57BL/6, Rosa-LSL-tdTomato (known as Ai14*), SMNΔ7 mice (FVB.Cg-Grm7Tg(SMN2)89Ahmb Smn1tm1Msd Tg(SMN2*delta7) 4299Ahmb/J, Stock No: 005025) were purchased from the Jackson laboratory. The mice were housed in a 12-hours light/dark cycle (light between 06:00 and 18:00) in a temperature-controlled room (22 ± 1 °C) with free access to water and food. Both male and female mice were used in this study. |
| Wild animals | This study did not involve wild animals. |
| Reporting on sex | The data for both male and female mice are described independently in this study. |
| Field-collected samples | None were used |
| Ethics oversight | All procedures were performed in accordance with protocols approved by the IACUC and Animal Resources Department of the Salk Institute for Biological Studies. |

Note that full information on the approval of the study protocol must also be provided in the manuscript.
